# EFFECT MODIFICATION OF AGE AND SEX BETWEEN AORTIC CALCIFICATION AND MORTALITY IN A COMMUNITY SAMPLE OF OLDER PATIENTS

**DOI:** 10.1093/geroni/igae098.0429

**Published:** 2024-12-31

**Authors:** Steven Horbal, Aleda Leis, Sven Holcombe, Carrie Karvonen-Gutierrez

**Affiliations:** University of Michigan, Ann Arbor, Michigan, United States; University of Michigan, Ann Arbor, Michigan, United States; University of Michigan, Ann Arbor, Michigan, United States; University of Michigan, Ann Arbor, Michigan, United States

## Abstract

Abdominal aortic calcification (AAC) is a useful cardiovascular risk assessor in opportunistic screening. As cardiovascular risk changes with increasing age, effective AAC use depends on understanding influential factors on AAC and mortality. This study evaluated modification of age and sex on the relationship between aortic calcification and mortality. This sample includes 6647 Michigan Medicine patients age ≥ 65 years, without cardiovascular disease, and who received an abdominal computed tomography scan between 1999-2022. AAC was elevated if calcification exceeded 19.26% of the aortic wall at the L3 vertebral level. Cox regression was used to assess AAC and mortality (adjusted for body mass index and Charlson Comorbidity Index) and stratified by age group (65-79, 80+ years) and sex. In the sample, 18.3% (n=1219) were 80+ years and the mortality rate was 38.0%. Those with elevated AAC had 99% higher mortality hazard than those with unelevated AAC [HR 1.99 (95% CI 1.66, 2.41)]. A statistically significant interaction was observed between age category, sex, and AAC status. Among patients 80+ years, elevated AAC was protective for mortality [HR 0.79 (95% CI 0.62, 0.98)]. Further, elevated AAC was associated with 35% lower mortality hazard in females age 80+ [HR 0.65 (95% CI 0.46, 0.92)] but was not significant in males age 80+. Important effect modification by age and sex exists on the relationship between AAC and mortality. This suggests the impact of AAC as a risk factor for mortality may decrease with age and be less important in 80+ year old females relative to males.

